# syntenyPlotteR: a user-friendly R package to visualize genome synteny, ideal for both experienced and novice bioinformaticians

**DOI:** 10.1093/bioadv/vbad161

**Published:** 2023-11-08

**Authors:** Sarah Quigley, Joana Damas, Denis M Larkin, Marta Farré

**Affiliations:** School of Biosciences, University of Kent, Canterbury CT2 7NJ, United Kingdom; The Genome Center, University of California, Davis, Davis, CA 95616, United States; Comparative Biomedical Sciences, Royal Veterinary College, London NW1 0TU, United Kingdom; School of Biosciences, University of Kent, Canterbury CT2 7NJ, United Kingdom

## Abstract

**Motivation:**

The rapid increase in the number of chromosome-scale genome assemblies has renewed interest in chromosome evolution studies. The visualization of syntenic relationships between genomes is a crucial initial step in the study of chromosome rearrangements and evolution. There are few tools available that serve this purpose, and they can be difficult to learn. Moreover, these tools are limited in the number of species comparisons that can be visualized and the size of chromosome rearrangements identified. Thus, the development of novel visualization tools is in strong need.

**Results:**

Here, we present syntenyPlotteR, an R package developed to visualize homologous synteny blocks in a pairwise or multispecies manner. This package contains three functions that allow users to generate publication-quality representations of syntenic relationships easily and quickly between genomes of interest.

**Availability and implementation:**

SyntenyPlotteR can be installed from CRAN with the documentation found in https://farre-lab.github.io/syntenyPlotteR/.

## 1 Introduction

In recent years, a large number of species genome assemblies have been publicly released by large sequencing projects (e.g. Rhie *et al.* 2021), allowing for improved bioinformatic analysis of chromosome structure and content. While this opens new doors for understanding the rules of life, it also raises some difficulties, particularly those relating to the scalability of existing methodologies (Damas *et al.* 2021).

Most new genomes are assembled to chromosome-scale, with one contig or scaffold spanning a full chromosome or chromosome arm. Consequently, the study of chromosome evolution is undergoing a renewal of interest. Identification, characterization, and visualization of chromosome rearrangements are critical steps in comparative genome analysis and the inference of a genome’s evolutionary history (Damas *et al.* 2021).

Synteny visualization has commonly used a circular representation of the genomes, such as with the tool Circos (Krzywinski *et al.* 2009). However, this style of visualization hinders the clear identification of small syntenic blocks and intra-chromosomal rearrangements (e.g. inversions) when the compared genomes have several chromosomes, and its use requires a steep learning curve. In contrast, linear plots of syntenic regions can aid in the identification of intra- and inter-chromosomal rearrangements (e.g. fissions, fusions, and translocations). Numerous tools written in R, a highly used programming language in bioinformatics (R Core Team 2016), have been developed for linear genome visualization, such as karyoploteR (Gel and Serra 2017), but they are not designed to plot syntenic relationships between genomes. Instead, in recent years, packages specifically designed to plot synteny have become more popular, such as RIdeogram (Hao *et al.* 2020) and ShinySyn (Xiao and Lam 2022). Both tools require extensive programming experience and have limitations in the number of species that can be compared and the resolution of chromosome rearrangements that can be identified. As a result, there is a critical need for new and easy-to-use tools for visualizing syntenic relationships.

Here, we present syntenyPlotteR, an R package to visualize syntenic relationships between genomes. This package incorporates three styles of syntenic plots allowing users flexibility to easily and quickly generate publication-quality representations of the syntenic relationships between genomes of interest. SyntenyPlotteR is a simple R package employing the use of single-function calls and requiring only two input data types.

## 2 Features

**Figure 1.**
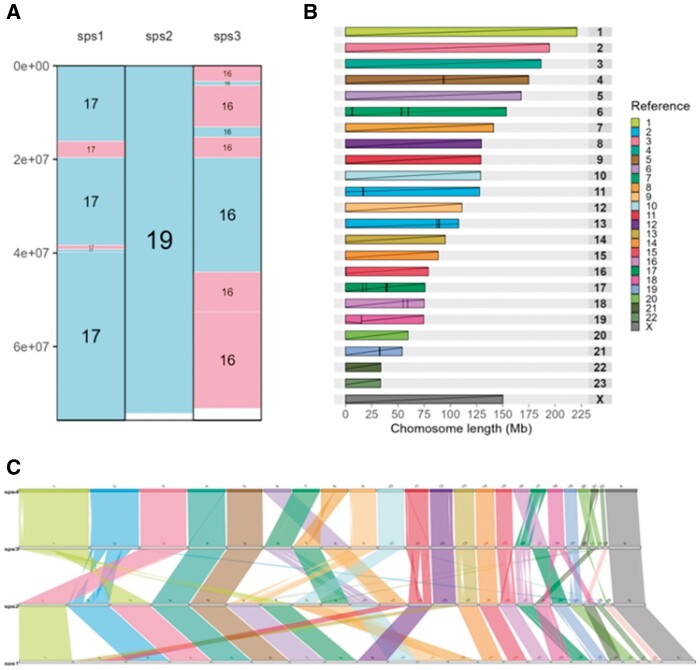
Three visualization styles available in syntenyPlotteR. (A) Evolution Highway style. Example dataset was created from a reduced group of alignments taken from human-lineage syntenic fragments in the reconstruction of a mammalian ancestor (Damas *et al.* 2022), where sps4 was compared to sps1, sps2, and sps3. Rectangles identify homologous synteny blocks (HSBs) where blue represents the same orientation (‘+’) as in the reference genome, and pink represents the opposite orientation (‘–’). Numbers in the blocks are chromosome numbers in the target species’ genomes. (B) Chromosome painting style. The target species chromosomes are represented by individual rectangles and the HSBs within are coloured according to their homologous reference chromosome. Diagonals inside the rectangles show the relative orientation of the HSBs in the two genomes, with upward lines representing the same orientation and downward lines representing opposite orientations. Vertical bars identify the boundaries of HSBs. (C) Linear comparison style. Reference chromosomes are colour-coded and coloured ribbons indicate homologous regions between the genomes. The chromosomes are ordered from reference at the top to target at the bottom. A twist in the ribbon indicates an inversion.

The new syntenyPlotteR is built as a package for R, a commonly used programming language in bioinformatics (R Core Team 2016). Two file types are required as input: (i) alignment files containing the pairwise syntenic regions between the reference and target species, and (ii) a file containing the lengths of the chromosomes for all species in the alignment files. Three visualization styles are included in the package: (i) Evolution Highway, (ii) Chromosome painting, and (iii) Linear comparison. Using a reduced dataset from previous publications (Damas *et al.* 2022), we demonstrate how these visualization styles show chromosome rearrangements between species’ genomes (Fig. 1).

### 2.1 Evolution highway visualization

Evolution Highway style mirrors previous publications (Kim *et al.* 2017, Farré *et al.* 2019), where syntenic data from several species’ genomes are compared to a common reference genome. As such, this style allows for the visualization of syntenic relationships between the reference and one or more species, with an unlimited number of species that can be plotted at a time. Each reference species chromosome is depicted as a standalone graph, with the default blue and pink block colours defining homologous syntenic blocks (HSBs) in the target species in the same (‘+’) or opposite (‘–’) orientation relative to the reference chromosomes, respectively (Fig. 1A). Regions of the chromosomes which are not aligned or assembled are represented as blank regions in the plots. Numbers within the blocks depict chromosome, scaffold, or contig numbers in each of the target species. This style is particularly suitable to detect intra-chromosomal rearrangements by identifying changes in block colours within the same target species chromosome. Inter-chromosomal rearrangements can also be detected by inspecting the numbers within the HSBs. The colours of the blocks are customizable by the user.

### 2.2 Chromosome painting visualization

Chromosome painting style plots have been used to visualize pairwise syntenic relationships between two genome assemblies of the same species (Rhie *et al.* 2021), different species, or compare a reconstructed ancestral genome with that of an extant species (Farré *et al.* 2019). In this visualization style, each target chromosome is depicted as a rectangle, and each HSB is painted according to its reference chromosome homologue (Fig. 1B). Each HSB is traversed by a diagonal line, such that completely syntenic chromosomes are traversed by a single line from bottom left to top right. Changes in the orientation of HSBs are shown by a diagonal line from top left to bottom right. The position of the diagonal line on the *y*-axis depicts the relative position of this HSB in the reference species’ chromosome. This visualization style assists in the identification of both intra- and inter-chromosomal rearrangements between two genome assemblies. As with the Evolution Highway plots, regions that either do not align or were not assembled in the comparative species are depicted as uncoloured regions of the reference chromosomes.

### 2.3 Linear comparison visualization

This style aims to facilitate the visualization of small intra- and inter-chromosomal rearrangements between a set of genomes. Comparison of linear genomes is achieved by plotting reference and target genomes connected by ribbons depicting their syntenic relationships (Fig. 1C). The reference chromosomes are colour-coded, and ribbons plotting the syntenic relationship follow the same colour scheme; while the target chromosomes are plotted in grey. Intra-chromosomal rearrangements are shown by a twist in the ribbon. This style is particularly useful to simultaneously identify intra- and inter-chromosomal rearrangements in all chromosomes of a linear genome comparison. There is no limit to how many genome comparisons can be plotted in the graph, provided they are input to the function in order from the first pairwise alignment file (at the top of the plot, Fig. 1C) to the final pairwise alignment (at the bottom of the plot, Fig. 1C). The colours are also customizable by the user if desired.

### 2.4 Additional functions

To facilitate the use of syntenyPlotteR, we also provide a function to reformat input data from commonly used syntenic file formats, such as those produced by inferCARs and DESCHRAMBLER (Kim *et al.* 2017).

## 3 Conclusions

We developed syntenyPlotteR, an R package specifically designed to plot syntenic relationships between genomes, allowing the clear identification of both inter- and intra-chromosomal rearrangements. SyntenyPlotteR is hosted in CRAN, more information and examples are provided within the vignettes and the R help() function. Details of the tool and manual can be accessed in https://farre-lab.github.io/syntenyPlotteR.
